# Supplementary material for: Are carbon emissions trading and green financial instruments synergistic? -Comprehensive quantitative research based on content analysis
Source: PLoS One. 2024 Mar 7;19(3):e0298601. doi: 10.1371/journal.pone.0298601 (PMC10919601; doi:10.1371/journal.pone.0298601)
Supplement: S1 Data — (ZIP) [file pone.0298601.s001.zip › Data/地级市Z_F工作报告.zip!/╕╩╦α/└╝╓▌╩╨/└┤╫╘╣1⁄2╓┌║┼í╛┬φ┐╦╩2╛▌═°í┐_2011.docx]

各位代表：

现在，我代表市人民政府向大会作报告，请连同《兰州市国民经济和社会发展第十二个五年规划纲要（草案）》一并审议，并请各位政协委员和列席人员提出意见。

“十一五”时期，是改革开放以来兰州经济社会发展最好最快的时期。五年来，在省委、省政府和市委的正确领导下，全市各族人民坚持以邓小平理论和“三个代表”重要思想为指导，深入贯彻落实科学发展观，抢抓西部大开发政策机遇，不断解放思想，完善发展思路，积极应对全球金融危机等困难和挑战，“十一五”发展的各项目标任务顺利完成。

综合经济实力显著增强。全市生产总值、地区性财政收入、地方财政总收入年均分别增长 12%、25.81%和27.4%，人均生产总值达到32900元，年均增长11%。全社会固定资产投资累计完成2255亿元，是“十五”时期的2.1倍，年均增长20.5%。配合国家和省上实施了兰渝铁路、兰州铁路枢纽、国家石油储备基地、输油气管道等一批重大项目。建成了兰州石化70万吨乙烯、榆中钢厂、甘肃会展中心、柴家峡水电站等重点项目。工业增加值和社会消费品零售总额分别翻一番，达到398亿元和546亿元，年均增长13.6%和16.3%。非公有制经济增加值由171.57亿元提高到430亿元，年均增长20.2%，占生产总值的比重达到40.2%。三次产业比例调整为2.9∶48.4∶48.7。全面完成节能减排任务，万元生产总值能耗下降20%。

城乡基础设施条件明显改善。五年城乡建设累计投入627亿元，完成北滨河路西延、天水路改扩建、南河道整治、城市出入口改造、金城关古建筑群等一批重大基础设施项目，加快实施供水、供电、供气、供暖等公用设施建设，城市综合功能进一步完善。大力推进小城镇和新农村建设，着力实施农田水利、电力通讯、交通能源等基础设施和社会事业项目，建设新农村示范村101个，新建农村公路4800公里，沼气3万户，累计解决54万人安全饮水问题。城中村改造取得明显成效，建成和开工建设村民安置房210万平方米，近10万城中村农民转为城市居民。城乡一体化建设积极推进，城镇化率由59%提高到62%。生态建设和环境保护取得重大进展，全市森林覆盖率由9.25%提高到12.21%，城区人均公共绿地面积达到8.93平方米。实施了清洁能源改造“123计划”等重点环境治理工程，空气污染综合指数由3.18下降到3.01，城区污水处理率达到95%以上，黄河兰州段功能区和饮用水源地水质达标率保持在100%。城市环境卫生集中整治取得明显成效，城市面貌有了大的改观。

改革开放取得重大突破。国企改革“393”攻坚任务全面完成，239户企业进行了改制，8.3万多名职工置换国企身份，209户企业实现资产重组。新一轮政府机构改革全面完成，行政管理体制和审批制度改革稳步推进，行政审批事项由1068项减少到328项。投融资体制改革成效显著，成功发行企业债券15亿元。文化体制改革深入实施，20家文化单位完成转企改制，兰州大剧院文艺创作和演出实力明显增强。农村土地流转、水管体制和集体林权制度改革逐步深化。科技、教育、财税、金融、医疗、住房以及社会管理等方面的改革全面推进。对外开放进一步扩大，五年累计引进国内资金652.4亿元，合同利用外资3.2亿美元。引进世界500强在内的大企业 30多家，外商投资企业发展到254户，外贸进出口总额达到37.6亿美元。

社会各项事业全面发展。五年累计用于社会事业的投入285亿元，是“十五”时期的2.2倍。科技创新和支撑能力进一步增强，科技进步对经济增长的贡献率达到55%，高于全国平均水平，兰州被列为国家创新型试点城市。各级各类教育协调发展，教育布局调整优化，城乡办学条件明显改善。“文化兰州”建设深入推进，中国秦腔博物馆、非物质文化遗产博物馆、国学馆、地震博物馆等一批重点文化设施相继建成。城乡卫生基础设施进一步完善，完成66个乡镇卫生院和 772个村卫生所标准化建设。全民健身活动和竞技体育蓬勃开展。统计、审计、物价、工商、质监、食品药品监管、广播电视、司法、民族宗教、人口与计划生育、残疾人救助、第六次人口普查等工作取得了新的成绩。“平安兰州”建设深入推进，安全生产形势持续好转，社会大局和谐稳定。民主法治和精神文明建设不断加强，“五城联创”工作取得积极成效，先后荣获省级历史文化名城和卫生城市、全国创建文明城市工作先进城市称号，“双拥模范城”建设实现“六连冠”。

人民生活水平大幅提高。城市居民人均可支配收入净增5471元，农民人均纯收入净增1847元，年均分别增长10.4%和10.9%。开工建设各类保障性住房300多万平方米，城镇居民人均居住面积达到15平方米。参加养老、失业、医疗、工伤和生育保险的总人数达到200万人。城镇居民基本医疗保险参保率达到80%以上，新型农村合作医疗参合率达到94%以上。低收入群体和困难群众生活得到保障，城乡低保实现了动态管理下的应保尽保，五年累计发放低保金、困难群体生活补贴、住房补贴、取暖补贴、交通补贴超过15亿元。全市贫困人口由23.98万人减少到17.6万人，农村贫困面下降5.8个百分点。为民兴办近100件实事，解决了一大批群众关心的热点难点问题。

2010年，是“十一五”规划的最后一年。我们紧紧抓住国家深入推进西部大开发、支持甘肃经济社会发展和省委新的区域发展战略出台实施等重大机遇，认真落实市委“1355”总体发展思路、“打好六大战役、实现六个突破”的总体部署和“再造兰州”战略，突出“抓发展、治污染、拓空间、畅交通、强管理、提效能、保民生、促和谐”等重点工作，圆满完成了市十四届人大五次会议确定的各项目标任务。

一是经济平稳快速增长。预计全市生产总值突破1000亿元，达到1100亿元，增长12%以上；地区性财政收入突破300亿元，达到303亿元，增长18.91%；地方财政总收入突破100亿元，达到 127.66亿元，增长35.13%。农业优势特色产业发展壮大，推广全膜双垄栽培面积46万亩，粮食产量达到40万吨，实现农业增加值32亿元，增长5%。强化工业经济运行调控服务，实现工业增加值398亿元，增长12%左右。完成全社会固定资产投资660亿元，增长30%。实现社会消费品零售总额546亿元，增长18.5%。实现旅游收入63.5亿元，增长68.4%。金融机构各项存款余额、贷款余额和居民储蓄存款余额分别增长21%、32%和20%。城市居民人均可支配收入达到14000元，增长 10%；农民人均纯收入达到4560元，增长14%，是近年来增幅最高的一年。单位生产总值能耗下降4%左右，二氧化硫和化学需氧量分别下降2%和6%。

二是项目建设成效显著。紧紧抓住国家出台支持甘肃经济社会发展等政策机遇，全方位、宽领域、深层次凝炼项目，在多元支柱产业、城乡基础设施、环境治理、民生和社会事业等方面凝炼项目771项，计划投资4400多亿元。全市集中实施重大项目100项，总投资797.81亿元，完成投资252.6亿元。争取实施国家扩大内需项目289项，完成投资29.03亿元。

全力支持区域性重大项目建设，蓝星“1318”一期、金川科技园镍钴锰生产线、连铝26万吨电解铝、方大炭素堆内构件、兰州南和连城330千伏输变电等项目建成投产，兰渝铁路、兰新铁路第二双线、兰州铁路货运枢纽等项目进度加快，兰州石化公司催化汽油加氢装置、吉利汽车扩能改造、交大真空镀膜、新区高铁、中川机场改扩建等项目开工建设，与省交通厅签署了南绕城高速等4条公路省市联建项目。积极推进项目申报工作，加大跑省进京力度，先后到20多个国家部委汇报衔接项目，促进了城市轨道交通、核热电联产、生物医药基地、地质灾害防治等项目的争取，92个项目列入国家、省上“十二五”规划和专项规划。大力开展招商引资，组织了赴粤闽、江浙、港澳等招商考察活动,举办了第十六届兰洽会，签约各类国内合同项目269项，引进资金到位235.8亿元。创新项目管理方式，在重大项目建设和重点工作中推行“指挥部模式”，开展“百日会战抓落实、百日攻坚抓项目”活动，加强现场协调和督促检查，加快了项目实施进度。

三是破解发展难题初见成效。围绕强化“中心带动”作用，深入研究经济社会发展制约因素，一批突出瓶颈难题初步得到解决。针对城市发展空间受限难题，推进实施“再造兰州”战略，全面启动兰州新区规划建设，组建新区管理机构，开展规划设计国际招标，编制完成总体规划纲要，一批基础设施项目启动建设，与11家金融机构签订了金融授信合作协议。立足产业支撑不足实际，积极研究培育发展多元支柱产业，深入开展调查研究，制定出台《关于发展培育多元支柱产业的意见》，提出构建“3+8”板块经济体系，两个国家级开发区增容扩区启动实施，八个县区产业园区正在积极谋划，每个产业发展的实施方案基本确定。着力解决环境污染治理难题，开展大气污染成因及防治对策研究，启动“环境污染治理行动计划”，城区污水全收集全处理工程基本完成，雁儿湾、西固、盐场污水处理厂建设进展顺利，兰州被环保部确定为大气污染防治试点城市。大力实施“畅交通”工程，坚持集中整治、强化管理和完善路网三位一体，制定城市智能交通管理方案，实施广场西口等枢纽型什字和17个路口交通综合优化改造，启动市区出入口停车场建设，深入开展公交车、出租车行业综合整治，探索机动车尾号和过境大车限行等措施，交通拥堵问题得到一定缓解。

四是城乡规划建设管理进一步加强。成立城乡规划委员会，开展城市空间、综合交通两大战略研究，完成第四轮城市总体规划大纲和第一版城乡统筹总体规划，修编完善了和平地区、西站地区、黄河风情线优化提升、银滩文化名人岛等专项规划，城区控详规划覆盖率达到90%以上。集中实施城市路、桥、管、网等项目122项，100条小街巷整治等项目全部建成，南山路、庙滩子地区整体改造、西热东输管网等项目加快推进，深安、金雁、元通三座黄河大桥及七里河大桥、中山桥加固维修、北环路一期等项目开工建设，新区快速通道、城市轨道交通等项目取得重大进展。加快城乡统筹发展和新农村建设，编制新农村村庄规划200个，2个统筹城乡发展试点县区和10个试点镇建设取得积极进展，实施了农村路网、农田水利、产业发展、农民新居、环境整治等26个重点项目，建成农村沼气池9000座，新建改造农村公路1000多公里，61个城中村实现撤村建居。启动市级万米单元网格精细化管理平台。大力开展违法建设、广告管理和环境卫生整治活动，拆除各类违法建筑15.7万平方米，解决“半拉子”工程和“断头路”问题初见成效。

五是民生热点难点问题逐步解决。为民兴办的20件实事全面完成。积极推进国家级创业型城市创建工作，培训各类劳动力3.76万人，安置困难群体就业近1万人，城镇新增就业5.64万人，城镇登记失业率控制在4%；输转劳动力32.5万人次，创劳务收入27.38亿元。社会保障覆盖面不断扩大，养老、失业、医疗、工伤、生育参保人数分别达到32.25万人、57.31万人、78.68万人、41万人和34.6万人，新型农村合作医疗参保人数达到112万人。公共卫生服务均等化重点工作有效推进，启动实施国家基本药物制度，群众看病就医费用明显降低。建成市儿童福利院、市残疾人托养就业康复中心。加强保障性住房建设，新开工经济适用房126.5万平方米、廉租房21万平方米、棚户区改造130万平方米，完成农村危旧房改造10368户。全面推动解决房产证历史遗留问题，为2334户城市居民登记发证。实施中小学校舍安全工程，239所汶川地震受损学校恢复重建项目全部开工建设，建成102所农村寄宿制学校，发放义务教育“两免一补”资金1.36亿元，群众反映强烈的教育热点问题得到缓解。制定出台稳定物价具体政策，启动市场价格应急预案，及时为困难群众发放临时物价补贴，居民消费价格基本稳定。

六是社会保持和谐稳定。加大社会治安综合治理力度，技防建设覆盖面达到86.3%，群众对社会治安的满意度有所提高。扎实开展矛盾纠纷排查化解和“两访”治理活动，解决了一批重点疑难信访案件。食品药品放心工程积极推进，开展食品药品安全专项整治行动，查处了一批非法经营和违法案件，创建规范化药房100家，食品药品放心消费示范门店100家。深入开展“安全生产年”活动，煤矿、道路交通、消防火灾等领域安全生产状况进一步改善。完成红古区29家小煤矿关闭任务，全面彻底解决窑街矿区历史遗留问题。应急管理工作进一步改进，妥善处置了兰钢幼儿园食物中毒、窑街煤电公司瓦斯突出等突发事件。创建全国文明城市工作深入推进，在中央文明委开展的全国公共文明指数测评中，我市列全国省会城市第16位，西北省会城市第1位。

七是行政服务水平明显提高。以强化执行力和优化发展环境为目标，在全市集中开展了“行政效能建设年”活动，广大干部的效能意识普遍增强，行政执行力得到提高。完善政府决策规则和程序，重大工作决策、重点项目招投标、大额度资金使用等事项一律集体决策。实施市级政府部门机构改革，优化和规范职能部门设置，在规划、国土等部门实行了“网上收件、限时办结、上下互联、在线监督”的网络化审批。创新行政管理手段，全面启动“数字兰州”建设工程，完成政府门户网站改版，推行网上办公和手机办公，初步实现市政府常务会议无纸化召开。借助网络问政于民、问计于民、问需于民，办理人民网、政府网留言2000多条。加强政务督查工作，建立任务管理、联动督查、考核通报、调研服务等机制，督查重大决策和重点工作800多项，办理省市人大代表和政协委员议案、提案和建议1084件。重点工作和重大项目向社会公开承诺，接受广大群众的监督，促进了工作落实。各级干部深入基层现场办公，在一线研究解决疑难问题，形成了思想务实、工作落实、作风扎实的氛围。

各位代表，“十一五”时期兰州经济社会发展取得的成就，是在省委、省政府和市委正确领导下，全市广大干部群众开拓创新、攻坚克难、奋力拼搏的结果，是市人大、市政协和社会各界监督支持的结果。在这里，我代表市人民政府，向各位代表、政协委员和全市各族人民，向各民主党派和工商联、各人民团体、社会各界人士，向离退休老同志，向驻兰解放军指战员、武警官兵和公安民警，向各级新闻媒体和广大网友，向所有关心、支持和参与兰州现代化建设的同志们和朋友们表示崇高的敬意和衷心的感谢！

“十一五”发展取得的成就，为“十二五”发展奠定了坚实基础，也积累了许多宝贵的经验。我们体会到，兰州要实现经济社会全面协调可持续发展，必须不断深化对市情的认识，坚持在继承中创新、在创新中发展，把握政策机遇，不断完善发展思路；必须充分发挥省会中心城市的区位、科教、人才、文化、商贸等综合优势，以项目建设为支撑，调整经济结构，培育发展多元支柱产业，从整体上增强经济实力；必须围绕提升城市影响力和带动力，优化城市空间布局，加强基础设施建设，完善城市服务功能，综合治理“城市病”，塑造城市特色和形象；必须把保障和改善民生作为一切工作的出发点和落脚点，在抓好经济发展的同时，协调推进社会事业发展，切实为群众办实事办好事，让人民群众共享改革发展成果；必须坚定不移地推进改革开放，善于用世界眼光和战略思维分析解决问题，积极学习借鉴外地先进经验，在工作中敢闯敢试、开拓创新、破解难题；必须大力发扬真抓实干精神，提高行政效能，优化发展环境，紧紧依靠广大干部群众，形成人心思进、创业实干、共谋发展的合力。这些既是“十一五”以来的经验总结，也是我们在今后工作中应当坚持的重要原则。

回顾过去五年的工作，我们也清醒地看到，我市经济社会发展中长期积累的矛盾和问题还很多，有些还比较突出。一是发展速度还不快，综合经济实力不够强，中心带动作用发挥不充分；二是传统产业所占比重大，高附加值的新兴产业发展不足，产业集中度不高；三是自我发展能力不高，生产要素集聚程度低，市场化融资渠道比较单一，新的经济增长点不明显；四是区域性科技、教育、人才资源整合和制度创新不足，转化利用不充分，潜在优势没有完全发挥出来；五是城乡基础设施欠账较多、功能薄弱，交通拥堵、大气污染等问题比较突出；六是社会各项事业发展仍然不足，就业矛盾比较突出，社会保障体系尚不完善；七是政府职能和机关作风还不适应市场经济发展的要求，行政效能和政府执行力有待进一步提高。我们必须围绕研究解决这些问题，在加快发展中破解难题，在破解难题中加快发展。

《兰州市国民经济和社会发展第十二个五年规划纲要（草案）》说明

“十二五”时期，是全面建设小康社会的关键时期，也是我们加快推进兰州率先跨越发展的重要时期。市委十一届八次全委（扩大）会议通过的《中共兰州市委关于制定国民经济和社会发展第十二个五年规划的建议》，围绕落实全省跨越式发展的部署要求，准确把握发展变化的市情特点，提出了未来五年我市经济社会发展的指导思想、奋斗目标和主要任务。根据市委《建议》，市政府制定了《兰州市国民经济和社会发展第十二个五年规划纲要（草案）》，已提请本次大会审议。下面，我就《纲要（草案）》作几点说明。

（一）“十二五”规划《纲要（草案）》的编制过程

“十二五”规划编制工作是在市委领导下进行的。2009年初，市政府组织有关方面，对全市经济社会发展面临的28项重大问题进行专题研究，市人大、市政协也就“十二五”发展的一系列问题组织开展调研，为编制《纲要（草案）》提供了重要基础。市委十一届八次全委（扩大）会议后，市政府根据市委《建议》精神，深入研究和编制“十二五”规划纲要，组织各方面专家进行咨询论证，在全市范围内广泛征求意见。《纲要（草案）》编制中，坚持贯彻中央和省上精神与兰州实际相结合，牢牢把握省委、省政府对兰州工作的要求，立足市情实际有针对性的贯彻落实，使之转化为具体项目和措施，增强可操作性；坚持发展思路的连续性与创新性相结合，根据“十二五”时期的新形势和新任务，对市委“1355”总体发展思路和“再造兰州”战略进行细化和拓展，与“十一五”规划和全面建设小康社会目标作了有效衔接；坚持立足当前和谋划长远相结合，在全力保持当前经济较快增长的同时，充分考虑率先跨越和可持续发展，力求夯实发展基础，增强发展活力。《纲要（草案）》的编制过程，是深化市情认识、总结发展经验、把握未来趋势的过程，是发扬民主、集思广益、科学决策的过程，是增强信心、凝心聚力、共谋发展的过程。

（二）“十二五”时期经济社会发展的总体要求和目标任务

《纲要（草案）》根据市委《建议》，提出“十二五”时期兰州经济社会发展的总体要求是：围绕率先跨越发展，立足发挥中心带动作用和提升城市影响力，以科学发展为主题，以加快转变经济发展方式为主线，以显著增加城乡居民收入为目标，以改革创新为动力，认真贯彻落实中央和省上一系列重大决策部署，深入实施“1355”总体发展思路和“再造兰州”战略，加快推进兰州新区、高新区、经济区开发建设，加快培育发展多元支柱产业，加快基础设施建设步伐，加快城乡一体化进程，加快各项社会事业发展，经过五年的努力，把兰州建成国家重要的战略性石化产业基地和有色冶金、装备制造、新能源、新材料及生物医药产业基地，西部地区具有重要影响的特大型现代化中心城市和西陇海兰新经济带的重要支点城市，西北交通枢纽和物流中心，新一轮西部大开发的重要增长极，全省跨越式发展的率先发展区。

《纲要（草案）》提出“十二五”时期经济社会发展的主要预期目标是，在全面协调可持续发展的前提下，确保“三个高于”、力争“七个翻番”、做到“三个控制”、实现“五个提升”。“三个高于”，即全市生产总值增速明显高于“十一五”时期，高于全省平均水平，高于西部省会城市平均水平。“七个翻番”，即生产总值、固定资产投资、社会消费品零售总额、地区性财政收入、城市居民人均可支配收入、农民人均纯收入、引进国内外资金等主要经济指标，在“十一五”发展基础上实现翻一番。“三个控制”，即城镇登记失业率控制在4%以内，人口自然增长率控制在5.5‰以内，节能减排控制在国家和省上下达的指标以内。“五个提升”，即城市综合经济实力大幅提升，城市影响力和带动力大幅提升，社会公共服务水平大幅提升，城乡居民生活水平大幅提升，社会和谐文明程度大幅提升。这些目标是综合考虑各方面因素，根据需要和可能提出的，经过努力是能够实现的。

（三）“十二五”时期经济社会发展的战略任务和重点工作

《纲要（草案）》提出“十二五”时期经济社会发展的主要任务是，统筹兼顾，集中力量，攻坚克难，在七个方面实现率先跨越：一是举全市之力，高起点规划，高标准建设，高效率推进，强化基础建设，突出产业带动，优化空间布局，健全开发机制，促进要素聚集，在兰州新区建设上实现率先跨越；二是坚持一手抓传统产业改造升级，一手抓战略性新兴产业培育发展，转变发展方式，优化经济结构，增强经济发展的内生动力，在培育发展多元支柱产业上实现率先跨越；三是坚持旧城改造和新城建设两轮驱动，全面加强城市道路、黄河桥梁、公用设施、文体场馆、人居环境等建设，着力完善城市综合服务功能，在基础设施和环境建设上实现率先跨越；四是加快新农村和小城镇建设，大力实施高原夏菜、生态建设、农田水利、特色农产品加工、草食畜、旱作农业、土地流转和农村康居百万工程，全力推进一体化，在统筹城乡发展上实现率先跨越；五是顺应全市人民过上更好生活的新期待，进一步完善低保、医保、养老、法律援助等社会保障体系，基本建立覆盖城乡的社会保障和医疗卫生体系，着力解决好事关群众切身利益的民生大事，在保障和改善民生上实现率先跨越；六是继续深化重点领域和关键环节改革，消除制约发展的体制机制障碍，扩大对内对外开放，不断增强发展的生机和活力，在深化改革开放上实现率先跨越；七是推进物质文明、政治文明、精神文明、社会文明和生态文明协调发展，在构建和谐兰州上实现率先跨越。

各位代表，实施“再造兰州”战略，建设兰州新区，是市委、市政府贯彻落实国务院办公厅《关于进一步支持甘肃经济社会发展的若干意见》、省委新的区域发展战略和跨越式发展的新目标，拓展城市发展空间，发挥中心带动作用，作出的重大决策。“再造兰州”就是按照“跳出老城建设新区、跨越发展再造兰州”的战略构想，坚持“两轮驱动、三足鼎立、多元支撑、基础先行、全面推进”，通过5至10年的努力，在城区面积拓展和经济总量上再造一个兰州。兰州新区建设以秦王川地区为主战场，将坚持长远规划、分期实施与滚动发展相结合，重点发展战略性新兴产业以及石油化工、装备制造、新能源、新材料、生物医药、现代农业和现代物流、仓储等服务业。实施“再造兰州”战略，加快兰州新区建设，是我们“十二五”乃至今后更长时期发展的重中之重，是兰州率先跨越发展的希望和潜力所在。我们要认真落实省上《关于加快推进兰州新区建设的指导意见》，集中人力、物力和财力，全力以赴推动兰州新区开发建设，努力把兰州新区建成发展战略性新兴产业、高新技术产业和循环经济的集聚区，国家经济转型和承接东部装备制造业转移的先导区，“两型”社会和城乡统筹发展的示范区，生态建设和未利用土地综合开发的实验区，传统优势产业和现代服务业的扩展区，向西开放的战略平台，沟通全省和西北地区的重要交通枢纽和物流中心，深入实施西部大开发战略的重要经济增长极。

2011年的政府工作

2011年是“十二五”发展的开局之年，我们面临的机遇前所未有，挑战前所未有，总体上机遇大于挑战，发展充满希望。政府工作将全面贯彻中央和省委、省政府的一系列决策部署，围绕再造兰州、率先跨越的主线，按照市委十一届八次全委（扩大）会议精神和全市经济工作会议“拓空间、调结构、强基础、保增长、惠民生、促发展”的要求，突出“三区”建设、多元支柱产业培育、城市形象提升、城乡统筹发展、公共服务均等化、区域经济合作、行政效能建设等重点工作，着力解决就业、就学、就医、住房、社保、物价等民生问题，保持经济社会平稳较快发展，确保实现“十二五”发展的良好开局，迈出率先跨越发展的坚实步伐。

按照率先跨越发展的总要求，2011年经济社会发展的主要预期目标是：全市生产总值增长 14%以上；工业增加值增长15%以上；全社会固定资产投资增长30%以上；社会消费品零售总额增长17%；地区性财政收入增长15%以上；地方财政总收入增长15%以上；财政公共投入增长10%；城市居民人均可支配收入和农民人均纯收入分别增长10%以上和13%以上；城镇登记失业率控制在4%以内；居民消费价格涨幅控制在4%以内；节能减排完成国家和省上下达的目标。

（一）加快构建以兰州新区为核心的板块经济体系，培育新的经济增长极。把园区经济作为率先跨越发展的主要平台，紧紧围绕构建“3+8”板块经济体系，坚持集约用地、集聚发展，全方位加大规划建设力度，夯实率先跨越发展的平台和支撑点。

优先推动新区基础设施建设。坚持规划的龙头地位，高起点凝炼新区特色，在总体规划的指导下，完成控详规划和产业发展、基础设施、生态建设等专项规划。强化产业区、核心区主干路网等基础设施建设，实施供水、供电、供气、通信等配套设施，加快快速通道、舟曲灾后安置、行政服务中心、黄河引水工程等项目建设，抓好调蓄水库、自来水厂、污水处理厂、垃圾处理场站等项目。统筹规划建设金融、税务、学校、医院、酒店等公共服务设施，为产业发展和人口集聚提供配套保障。全年完成固定资产投资200亿元以上。

突出产业带动新区开发。按照产业布局规划，把省内企业出城入园、改造升级与央企战略重组结合起来，积极引进央企和国内500强企业成片综合开发，推动中石油化工产业园、蓝星新材
